# Measuring experiences and concerns surrounding contraceptive induced side-effects in a nationally representative sample of contraceptive users: Evidence from PMA Ethiopia

**DOI:** 10.1016/j.conx.2022.100074

**Published:** 2022-03-19

**Authors:** Linnea A. Zimmerman, Dana O. Sarnak, Celia Karp, Shannon N. Wood, Mahari Yihdego, Solomon Shiferaw, Assefa Seme

**Affiliations:** a Department of Population, Family and Reproductive Health, Johns Hopkins Bloomberg School of Public health, Baltimore, MD, United States; b PMA-Ethiopia, Addis Ababa University, Addis Ababa, Ethiopia; c School of Public Health, Addis Ababa University, Addis Ababa, Ethiopia

**Keywords:** Contraceptive side-effects, Contraceptive-induced menstrual bleeding changes, Ethiopia

## Abstract

**Objective:**

Our objectives were to assess the prevalence of specific side-effects experienced by current and recent contraceptive users, describe patterns of side-effects that users were concerned about, and share measurement lessons learned.

**Study design:**

Data come from the PMA Ethiopia 2019 nationally-representative, cross-sectional survey. Our analytic sample included women who were current (weighted *n* = 2190; unweighted *n* = 2020) or recent (past 24 months; weighted *n* = 627; unweighted *n* = 622) users of a hormonal method or IUD. We provide descriptive statistics of the percentage of current/recent users who report currently/ever experiencing specific side-effects, not experiencing but being concerned about experiencing specific side-effects, and both currently experiencing and being concerned about experiencing specific side-effects. All analyses are stratified by method type (implant, injectable, pill) to explore variation by method.

**Results:**

Among current users, 648/2190 women (30%) reported experiencing any side-effect, while 252/644 (40%) of recent users reported ever experiencing any side-effect. Bleeding changes were reported most frequently and were higher among implant and injectable users. More recent users reported side-effects that were associated with physical discomfort, such as headaches, than current users. About one-third of current and recent users reported being concerned about at least one side-effect that they had not experienced, with about 15% of current and recent users reporting concerns about bleeding changes (307/2190 and 112/627, respectively) and concerns about physical discomfort (334/2019 and 98/627, respectively).

**Conclusions:**

While bleeding changes are common, users report a range of side-effects related to physical discomfort underscoring the need for comprehensive counseling. We highlight challenges in measuring side-effects using quantitative tools and pose recommendations for future research and measurement efforts.

**Implications:**

: Experiencing and fearing contraceptive-induced menstrual bleeding changes and physical discomfort, particularly headaches, is high among hormonal contraceptive and IUD users in Ethiopia. counseling that addresses an array of side-effects is needed. Additional research is also needed to disentangle the effect of experiencing versus fearing side-effects on contraceptive use.

## 1 Introduction

Fears of or prior experiences with contraceptive-induced side-effects are among the most frequently stated reasons for not using [1,2] and for discontinuing a contraceptive method while still wishing to delay or avoid pregnancy [3,4]. Qualitative evidence has documented a range of side-effects, both clinically-documented, such as changes to menstrual bleeding [5,6] and sexual pleasure [7,8], and wide-ranging fears, including increased risk of cancer, birth defects, and infertility [9], [10], [11], [12]. Despite substantial evidence regarding the influence of side-effects on contraceptive use, however, significant research gaps remain, particularly in low- and middle-income countries where unmet need for contraception remains high [13].

The primary sources of quantitative data on contraceptive use in low- and middle-income countries, specifically the Demographic and Health Survey (DHS), Performance Monitoring for Action (PMA), and the Multiple Indicator Cluster Survey (MICS), lack questions that identify specific side-effects [14], [15], [16]. Instead, reasons for non-use or discontinuation are indicated via non-specific response options of “side-effects” and “health concerns.” It is thus not possible to identify which side-effects are most prevalent or have the greatest influence on contraceptive dynamics at the population-level. It is also difficult to discern how experiences and/or concerns about side-effects differ by method. Though side-effect profiles for specific methods are readily available from clinical trials and medical guidance, few quantitative studies applying a social science lens have evaluated whether specific side-effects, either experienced or feared, are unique to particular methods [17,18]. Recent reviews exploring the impact of contraceptive-induced menstrual bleeding changes and changes to women's sexual experience resulting from contraceptive use have highlighted the importance of gathering data on specific side-effects, including by method [6,8].

Another research gap arises from challenges in quantitatively distinguishing side-effects that are experienced versus those that are feared. Women may attribute a range of potential outcomes—notably cancer, infertility, and poor birth outcomes [[9], [10], [11],^19^,^20^]—to use of a contraceptive method, despite substantial clinical evidence to the contrary. While qualitative evidence has found that fear of future infertility is particularly salient, few quantitative studies have documented the extent to which this concern exists among contraceptive users. Further complicating the “feared versus experienced” dichotomy of side-effects is the recognition that experience of some side-effects, such as contraceptive-induced menstrual bleeding changes, are linked to fears of infertility or cancer [6,10]. Understanding how experience of specific side-effects contributes to individual concerns and the spread of misinformation at the community-level is critical to designing contraceptive counseling and programming messages that are contextually relevant.

To address these limitations, Performance Monitoring for Action Ethiopia (PMA Ethiopia) included questions to assess experiences and concerns surrounding contraceptive side-effects among users. In this study, we aim to (1) assess the prevalence of specific side-effects experienced by current and recent hormonal users; and (2) describe patterns of side-effects that current and recent users were concerned about. Additionally, we aim to (3) share measurement lessons learned through examining overlap of reported experiences and concerns of specific side-effects.

## 2 Material and methods

### 2.1 Study setting

Ethiopia is a low-income, high fertility country at 4.2 children per woman [21]. Modern contraceptive use among all women rose from 5% in the year 2000 to 26% in 2020, however, side-effects and health concerns remain a major contributor to contraceptive non-use [2] and discontinuation while in need of a method [22,23]. The method mix is largely dominated by the injectable, at 58% of all modern users, followed by the contraceptive implant, at 30% of all modern users [24].

### 2.2 Data

We used data from the PMA Ethiopia cross-sectional survey, conducted from October-December 2019 [25]. PMA Ethiopia is a multistage cluster, nationally representative household survey of women age 15 to 49. Two hundred sixty-5 enumeration areas (EAs) were drawn with probability-proportional-to-size within strata. All women age 15 to 49 who were either usual members of the household or who slept in the household the night before were eligible, and if consented, interviewed by a trained female interviewer. A total sample of 8837 de-facto women were interviewed. Further information on the design of PMA Ethiopia is available from the study protocol [26]. PMA Ethiopia received ethical approval from Addis Ababa University, College of Health Sciences (Ref: AAUMF 01–008) and the Johns Hopkins University Bloomberg School of Public Health Institutional Review Board (FWA00000287).

Surveys included questions on the experience and concern of side-effects related to contraceptive use [27]. Questions were piloted in June 2019, including a detailed review by the field team for coherence, wording, and option choices. Cognitive interviews with rural and urban respondents to assess question comprehension and identify answer choices were also conducted. Women did not demonstrate any challenges in understanding questions.

### 2.3 Analytic sample

Our analytic sample was restricted to women who were current (*n* = 2020) or recent (past 24 months; *n* = 622) users of a hormonal method or Intrauterine Device (IUD) at the time of the survey (herein referred to as “all users”). Current versus recent use was mutually exclusive; a woman could either be classified as a current user (currently using a method) or a recent user (not currently using a method but used in the past 24 months). Current users who switched from another method in the previous 24 months are thus only included within current users. We explored recent use using both a 12- and 24-month window and included the 24-month window to maximize sample size. Hormonal contraceptive methods include implant, injectable, pill, and emergency contraception. We were unable to distinguish between hormonal and non-hormonal IUD users in the sample, however as only the Copper T380A is included in the Essential Medicine List for Ethiopia [28], this makes up the vast majority of IUD provision [29].

### 2.4 Measures

First, we assessed the proportions of side-effects that women currently or recently experienced while using hormonal contraceptives or IUDs via responses to the questions: “*What are the side-effects that you are currently experiencing/experienced while using the method?”*. Second, we assessed the distributions of concern for side-effects using the questions: *“Are there any side-effects that you are worried about experiencing while using this method, but are not actually experiencing?”* (current users) and *“What were the side-effects that you were worried about experiencing while using this method, but did not actually experience?"* (recent users). Response options were not read out loud and women spontaneously self-reported their responses. Though the questions were designed to mutually exclusive, as response options were not constrained, women could report the same side-effect for both questions. Response categories for experiences of and concerns for side-effects included a range of options generated from a literature review, a recent survey and qualitative study in Uganda (PMA2020 Uganda Round 7), and pilot-testing. The full list of response options is in Appendix A and B.

### 2.5 Analyses

We examined frequencies of our outcomes among the 3 most common contraceptive methods—implant, injectable, and pill—in addition to frequencies across all methods. Using the percent frequencies, we grouped experienced side-effects into 4 themes: “bleeding changes,” “discomfort,” “sexual experience,” and “other”, as shown in Table 1. Concerns were grouped into the same themes, with the addition of “fertility” and “cancer.” We tested for differences in the percentage of users who reported each side-effect by method type using design-based F-statistics to account for the complex survey design. Due to differences in the question wording, we did not test differences between current and recent users.

**Table 1:** Percentage of current and recent users who reported currently experiencing each side-effect, overall and by method; PMA Ethiopia 2019 Cross-Section

Current users						Recent users					
	Implant	Inject-able	Pill	*p*-value for diff across 3 methods	Total hormonal		Implant	Inject-able	Pill	*p*-value for diff across 3 methods	Total hormonal
**N**	**675**	**1114**	**158**		**2020**	**N**	**118**	**393**	**79**		**622**
	%	%	%		%		%	%	%		%
**Any side-effect**	**35.1**	**28.0**	**16.6**	0.00	**29.6**	**Any side-effect**	**50.0**	**39.0**	**35.5**	0.16	**40.4**
**Bleeding changes**	**25.6**	**22.0**	**12.4**	0.02	**22.3**	**Bleeding changes**	**33.2**	**29.7**	**18.6**	0.23	**29.2**
Less bleeding	12.5	14.6	2.8	0.00	12.9	Less bleeding	14.7	17.7	4.3	0.03	15.5
More bleeding	12.3	6.5	5.8	0.00	8.5	More bleeding	18.1	13.1	13.2	0.54	13.9
Irregular bleeding	11.1	5.4	8.5	0.00	7.5	Irregular bleeding	17.1	10.2	7.8	0.23	11.3
**Discomfort**	**21.0**	**15.6**	**3.7**	0.00	**16.8**	**Discomfort**	**39.4**	**24.9**	**20.8**	0.02	**26.6**
Headache	11.2	8.9	2.1	0.01	9.4	Headache	21.0	14.4	5.4	0.05	14.3
Weakness	5.3	2.9	0.7	0.00	3.7	Weakness	6.2	4.2	6.6	0.64	4.7
Backache	4.2	3.3	1.2	0.25	3.6	Backache	8.1	6.4	5.5	0.85	6.4
Abdominal pain	2.7	1.8	1.3	0.50	2.3	Abdominal pain	7.4	2.7	1.5	0.03	3.3
Cramping	2.5	2.4	0.0	0.45	2.3	Cramping	10.0	3.9	1.5	0.02	4.7
Nausea/vomiting	2.0	1.5	0.6	0.45	1.6	Nausea/vomiting	3.3	3.2	5.0	0.84	3.4
Insert pain	3.1	0.3	0.0	0.00	1.2	Insert pain	3.1	1.2	1.5	0.37	1.6
Infection	0.8	0.7	0.0	0.76	0.7	Infection	1.4	0.5	0.1	0.43	0.6
Diarrhea	0.7	0.3	0.0	0.57	0.4	Diarrhea	0.0	0.5	0.0	0.81	0.3
Lose weight	8.3	4.1	0.0	0.00	5.2	Lose weight	12.9	10.1	1.9	0.15	9.6
Gain weight	1.9	2.3	0.5	0.39	2.1	Gain weight	5.7	2.9	0.0	0.21	3.2
Acne	2.0	1.6	0.3	0.18	1.7	Acne	3.6	3.6	4.5	0.95	3.5
**Changes to sexual experience**	**2.3**	**1.7**	**0.1**	0.25	**1.9**	**Changes to sexual experience**	**2.8**	**3.6**	**0.4**	0.32	**3.0**
Low sex drive	1.7	1.4	0.0	0.61	1.5	Low sex drive	1.1	2.8	0.4	0.26	2.2
Less pleasure	1.5	1.1	0.1	0.29	1.3	Less pleasure	1.7	1.1	0.0	0.70	1.0
Vaginal dryness	0.8	0.4	0.0	0.58	0.5	Vaginal dryness	0.0	0.3	0.0	0.86	0.2
Partner	0.5	0.4	0.0	0.51	0.5	Partner	0.0	1.4	0.0	0.64	1.0
**Other**	**3.3**	**2.3**	**2.2**	0.73	**2.7**	**Other**	**8.2**	**5.7**	**8.0**	0.47	**6.2**
Mood swing	2.4	1.5	0.0	0.31	1.7	Mood swing	3.9	4.2	2.7	0.87	3.9
Other	1.1	0.8	2.2	0.41	1.1	Other	4.6	1.5	6.7	0.02	2.5

Respondents could report multiple side-effects, thus totals do not add up to 100%.

Category percentages (i.e., “Bleeding changes”) are not summative, but are calculated as the percentage of women who said yes to any one of the side-effects in the category. Bold indicates the percentage of women who reported at least one of the side effects listed within each group. For example, 25.6% of women using the implant indicated one or more bleeding related side-effects (less, more or irregular bleeding).

Finally, in exploratory analyses, we noted that some women who reported experiencing a specific side-effect also subsequently reported being concerned about the same side-effect. Given the phrasing of the question (*“Are there any side-effects that you are worried about experiencing while using this method, but are not actually experiencing?”),* we expected that women would not report experiencing and being concerned about, but not experiencing, the same side-effect. Therefore, to quantify the extent of this phenomena, we examined overlap in the percentage of women who reported simultaneously currently experiencing a side-effect and being concerned about, but not experiencing, the same side-effect. We calculated frequencies among implant, injectable, and all users; due to sample size limitations we could not explore this among pill users. Analysis was restricted to side-effects with a minimum of 15 observations.

## 3 Results

Sociodemographic characteristics of women in the sample are included in Appendix C. Table 1 shows frequencies of experienced side-effects, presented separately by current users versus recent users and by implant, injectable, pill, and all users.

Among current users, about one-third of women (29.6%) reported currently experiencing at least one side-effect; this varied significantly by method, with fewer pill users reporting side-effects. Bleeding changes were the most experienced side-effect. Reporting of each kind of bleeding change (less, more, and irregular) varied by method type. About 17% of all method users reported they were currently experiencing some form of physical discomfort, with headache being the most cited. Discomfort varied by method type, with a higher percentage of implant users reporting discomfort-related side-effects than either injectable or pill.

**Fig. 1:**
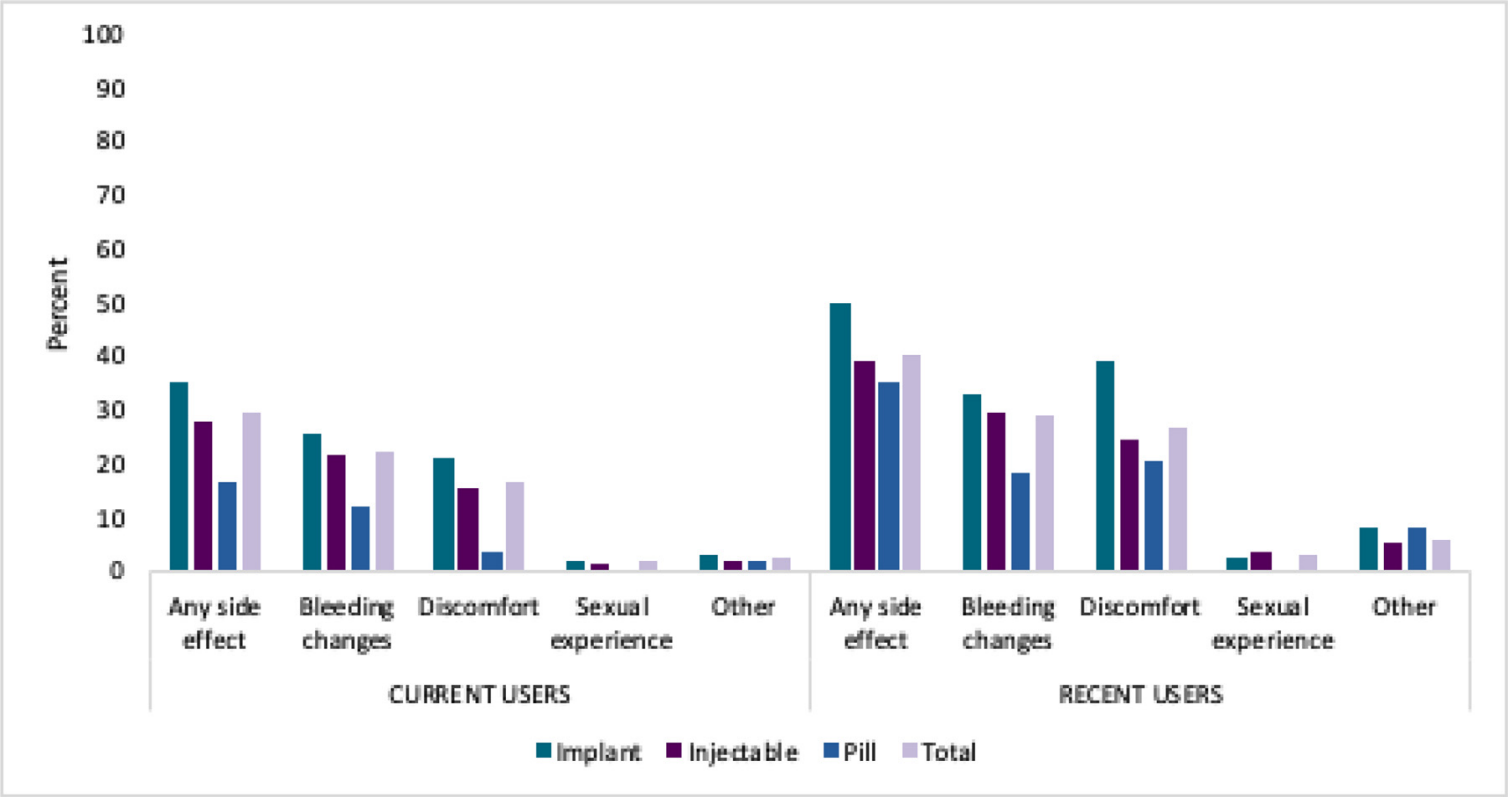
Percentage of current and recent users reporting experiencing each side-effect type by method; PMA Ethiopia 2019 Cross-Section.

Four in 10 recent users reported ever experiencing any side-effect (40.4%). In terms of menstrual bleeding change, the only variation by method was in reporting less-bleeding. Headaches were frequently reported among implant and injectable users. Among pill users, while more bleeding was the most commonly reported side-effect a range of discomfort related side-effects were reported (Fig. 1).

Table 2 presents frequencies of concerns surrounding side-effects. Among all method users, 27.9% of current users reported being concerned about at least one side-effect that they had not experienced, with little variation by method. Despite concerns being widespread across all methods, the most reported concerns varied by method type. Fertility-related fears, specifically delayed return to fertility and infertility, were reported by fewer than 5% of users were less common among implant users.

**Table 2:** Percentage of current and recent users who reported not experiencing but being concerned about each side-effect, overall and by method; PMA Ethiopia 2019 Cross-Section

Current users						Recent users					
	Implant	Inject-able	Pill	*p*-value for diff across 3 methods	Total hormonal		Implant	Inject-able	Pill	*p*-value for diff across 3 methods	Total hormonal
N	**675**	**1114**	**158**		**2020**	N	**118**	**393**	**79**		**622**
	%	%	%		%		%	%	%		%
Any side-effect	26.9	28.5	27.4	0.09	27.9	Any side-effect	30.1	34.9	22.7	0.17	33.0
**Bleeding changes**	**13.8**	**15.5**	**7.2**	0.12	**14.0**	**Bleeding changes**	**15.2**	**19.9**	**11.0**	0.27	**17.9**
Less bleeding	7.0	10.2	2.7	**0.03**	8.4	Less bleeding	9.7	13.3	2.1	0.07	11.4
More bleeding	8.8	5.4	4.7	0.08	6.4	More bleeding	8.5	7.4	9.0	0.89	7.6
Irregular bleeding	3.9	3.1	1.2	0.48	3.2	Irregular bleeding	2.6	5.3	4.2	0.53	4.8
**Discomfort**	**17.9**	**14.1**	**12.3**	0.15	**15.2**	**Discomfort**	**11.5**	**17.3**	**13.0**	0.29	**15.7**
Headache	5.8	5.9	4.5	0.86	5.8	Headache	5.6	6.8	3.3	0.63	6.0
Weakness	5.6	2.6	1.1	0.01	3.5	Weakness	3.8	4.9	3.9	0.86	4.4
Abdominal pain	1.6	1.5	0.7	0.78	1.5	Abdominal pain	3.5	2.2	2.5	0.78	2.6
Insert pain	2.7	0.8	0.0	0.02	1.4	Insert pain	2.7	0.5	0.0	0.12	0.9
Infection	1.8	0.5	0.9	0.07	1.1	Infection	0.0	0.6	0.0	0.68	0.4
Nausea/vomiting	0.7	1.0	1.2	0.83	1.0	Nausea/vomiting	0.6	2.9	0.7	0.08	2.2
Diarrhea	0.0	0.0	0.0	0.77	0.0	Diarrhea	0.0	0.0	0.0	NA	0.0
Weight change	7.3	5.1	2.4	0.13	5.6	Weight change	3.9	8.4	1.8	0.05	6.9
Acne	2.3	2.8	6.0	0.14	2.8	Acne	0.2	4.6	3.5	0.04	3.6
Lost in body	2.8	0.6	0.0	0.00	1.4	Lost in body	1.6	0.3	0.0	0.28	0.5
**Changes to sexual experience**	**1.8**	**0.8**	**0.0**	0.26	**1.2**	**Changes to sexual experience**	**0.0**	**1.6**	**0.0**	0.40	**1.1**
Low sex drive	1.0	0.5	0.0	0.44	0.7	Low sex drive	0.0	1.0	0.0	0.56	0.7
Less pleasure	0.9	0.3	0.0	0.28	0.6	Less pleasure	0.0	0.6	0.0	0.72	0.4
Vaginal dryness	0.5	0.5	0.0	0.79	0.4	Vaginal dryness	0.0	0.3	0.0	0.86	0.2
Partner	0.4	0.1	0.0	0.37	0.2	Partner	0.0	0.0	0.0	NA	0.0
**Fertility**	**4.5**	**8.4**	**6.0**	0.01	**6.9**	**Fertility**	**5.9**	**8.0**	**9.2**	0.66	**8.1**
Delayed fertility	2.7	4.2	1.4	0.06	3.6	Delayed fertility	3.6	3.3	3.7	0.98	3.4
Infertility	1.7	4.3	4.7	0.02	3.4	Infertility	3.7	4.6	5.5	0.82	4.9
Deformation of babies	0.3	0.7	0.0	0.57	0.5	Deformation of babies	0.0	0.5	0.0	0.74	0.3
**Cancer**	**1.1**	**2.2**	**2.4**	0.31	**1.8**	**Cancer**	**2.9**	**2.2**	**0.8**	0.59	**2.2**
Cancer/fibroids	0.5	0.8	0.0	0.57	0.7	Cancer/fibroids	1.1	1.1	0.0	0.75	0.9
Blood build up/impurities	0.6	1.4	0.0	0.23	1.0	Blood build up/impurities	1.9	1.1	0.8	0.72	1.3
Pills accumulate in body	0.0	0.1	2.4	0.00	0.3	Pills accumulate in body	0.0	0.0	0.0	NA	0.0
**Other**	**2.7**	**2.6**	**5.8**	0.20	**2.9**	**Other**	**7.2**	**2.8**	**0.8**	0.01	**3.5**
Mood swings	1.0	1.2	0.0	0.52	1.0	Mood swings	2.6	1.5	0.0	0.55	1.5
Increased hair growth	0.0	0.1	0.0	0.65	0.1	Increased hair growth	0.0	0.0	0.0	NA	0.0
Other	1.7	1.4	5.9	0.01	1.9	Other	4.8	1.4	0.8	0.01	2.0

Respondents could report multiple side-effects, thus totals do not add up to 100%.

Category percentages (i.e., “Bleeding changes”) are not summative, but are calculated as the percentage of women who said yes to any one of the side-effects in the category. Bold indicates the percentage of women who reported at least one of the side effects listed within each group. For example, 25.6% of women using the implant indicated one or more bleeding related side-effects (less, more or irregular bleeding)

**Fig. 2:**
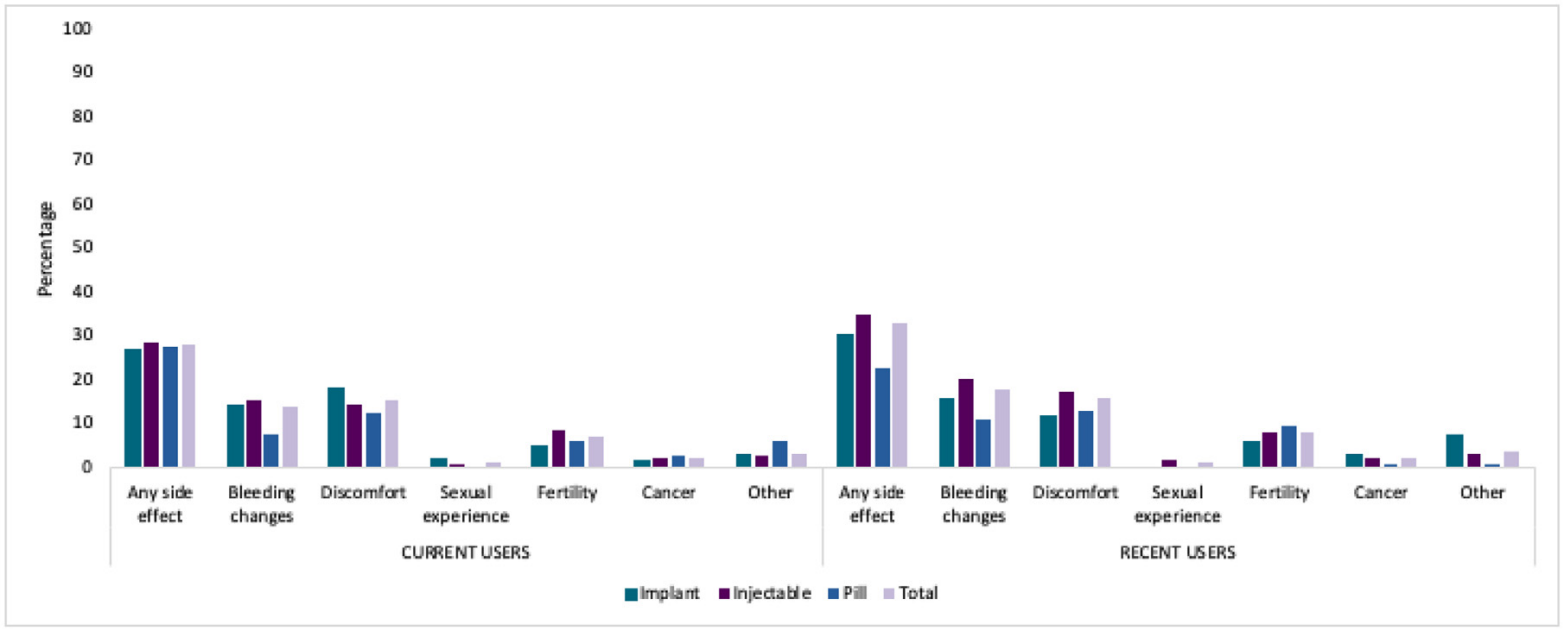
Percentage of current and recent users reporting being concerned about but not experiencing each side-effect type by method; PMA Ethiopia 2019 Cross-Section.

One-third of recent users reported being concerned about a side-effect that they did not experience. Among implant users, concern for either less or more bleeding and headaches predominated (Fig. 2). For injectable users, concerns about less bleeding and weight change were highest, and for pill users, concerns of more bleeding and infertility were most frequently reported. As with current users, fertility-related concerns were reported by fewer than 5% of users (except for 5.5% of pill users).

Finally, we examined whether women distinguished experiencing a side-effect from concerns surrounding a side-effect, as these items were meant to be mutually exclusive. Table 3 shows the percentage of women who stated that they were concerned about experiencing a side-effect, among those who reported currently experiencing that same side-effect. Large percentages of women reported experience and concern for the same side-effect. Differences were not significant by method.

**Table 3:** Percentage of women reporting that they were concerned about but had not experienced each side-effect, among women who reported currently experiencing the side-effect; PMA Ethiopia 2019 Cross-Section

	Implant		Injectable		Total hormonal	
	Percent	N	Percent	N	Percent	N
Side-effect feared						
Less bleeding	26.7	87	32.6	165	30.0	258
More bleeding	34.0	80	31.6	75	32.3	172
Irregular bleeding	23.2	68	30.1	66	24.9	151
Abdominal pain	20.9	15	24.5	18	19.0	41
Weight change	32.7	65	32.1	66	31.4	137
Acne	–	–	30.4	16	22.0	28
Headache	30.9	61	39.0	91	34.6	166
Weakness	25.7	33	13.1	29	17.9	67
Insert pain	17.1	19	–	–	18.4	25

## 4 Discussion

Contraceptive users reported an array of side-effects. While contraceptive-induced menstrual bleeding changes were common, both current and recent users reported a range of other side-effects that are more broadly related to pain and discomfort. Additionally, substantial minorities of women reported both experiencing and concern for the same side-effect, underscoring the challenge in measuring these concepts using quantitative surveys and the interplay of experiences and concerns.

Ours is the first study to assess the frequency of contraceptive-induced menstrual bleeding changes among a nationally representative group of women. While recent work has highlighted the important role of increased bleeding [5,10], the variety of contraceptive-induced menstrual bleeding changes highlights the need for counseling that covers a range of potential bleeding changes, such as those proposed by Rademacher and colleagues [30]. Additionally, we found that side-effects we grouped under “discomfort” were as prevalent, and for some method users, more prevalent than bleeding changes. As Jain et al. argue, expanding our understanding of a range of side-effects, the tolerability of different side-effects, and their effects on daily activities, is critical to improve counseling strategies and ultimately reduce dissatisfaction and discontinuation [31].

To inform future research efforts, we highlight relevant lessons and challenges. First, side-effect experiences and concerns did not seem to be mutually exclusive within this population. A sizeable portion of women (20-30%) who reported experiencing side-effects also said they were concerned that they would experience them. One explanation is that, despite question wording designed to explicitly separate these concepts, the intent of the question was unclear. Another potential explanation is that women who responded that they both experienced and were concerned about a specific side-effect did not conceptualize these 2 experiences as distinct. In either case, for many women, concerns over potential side-effects and experiences are interconnected. Qualitative studies have described fears as a byproduct of myths and misinformation [5,11], but recent research has attempted to disentangle these concerns, finding that fears are often a combination of personal experience and misinformation [9,10]. In our sample, we found considerable challenges in determining what side-effects women were concerned with, but did not experience, and encourage greater work in this area.

A second lesson arose from the low percentage of women who reported infertility-related concerns, which seems to contradict the preponderance of evidence that cites fears of future infertility as a major impediment to use [[9], [10], [11],^19^]. Few studies have assessed the prevalence of infertility-related concerns using quantitative data, making comparisons challenging. One study in Kenya found that across a range of contraceptive methods, 9% to 30% of current, recent, or never users believed a specific method could cause infertility [17]. Unfortunately, as we did not ask questions to non-users, we are unable to make a similar comparison. One explanation for our finding may be that even if women believe that contraception interferes with future fertility, if they have reached their desired number of children, they may be less concerned about this potential side-effect. Our question did not assess the prevalence of this belief, only whether women were concerned that they may experience side-effects, including fertility-related concerns. Understanding both the prevalence of this belief and whether it varies by use state (e.g., current, previous, or never user) are critical research endeavors, as they may guide effective messaging to dispel myths and improve counseling. Another explanation may be related to how women understand the term “side-effects” as it relates to questions about contraceptive use and fertility. Side-effects are generally defined using medical terminology and interpreted in the context of biomedical frameworks [32], but infertility is a complex phenomenon that may have perceived social and economic causes and consequences that complicate this conceptualization. Despite pilot testing, it is unclear if our question fully captured the perceived risk of future fertility impacts, and thus, a direct question may be a more useful alternative.

Our study has several limitations which justify further refinement and exploration. As noted above, the concept of side-effects is challenging to measure in a population-based survey. While researchers may have specific biomedical definitions of what constitutes a side-effect, women may consider a broader range of outcomes [32]. Additionally, what is reported as a side-effect may depend on information received from others; if a woman has not heard that a specific side-effect can be caused by contraception, she may not report it, or if she has heard frequently about a specific side-effect, she may be more likely to report it. Additionally, while changes to contraceptive bleeding present with clear physical symptoms, side-effects without an obvious physical symptom, such as a reduction to sex-drive, may be underreported. In this study, women were asked to self-report side-effects, none of which were clinically verified and many which may not be direct consequences of contraceptive use. This may lead to over-reporting of the true prevalence of side-effects in the population. Despite these limitations, studying what women themselves perceive and report as side-effects has value; women are not likely to make contraceptive decisions based on clinical verification, but lived experience [10]. It is critical to understand what outcomes women attribute to contraception to deliver effective counseling and communication messages.

An additional limitation is our inability to adequately explore the differences between current and recent users. We asked recent users whether they had *ever* experienced any side-effects, while we asked current users if they were *currently* experiencing any side-effects. This limits comparability between the 2 groups and likely explains why more recent users reported experiencing side-effects. This is an important comparison to make, however, and worth exploring in future research. As Machiyama et al. found in Kenya, if women who experience side-effects are more likely to discontinue and be dissatisfied, they may also be more likely to contribute to general negative attitudes towards contraception [17]. Additionally, recent users, as defined in our study, may be different than women who used a method in the past 24 months and switched, and who we considered current users. Women who experienced side-effects may be more likely to stop use altogether rather than switch methods [33], and thus higher rates of side-effects may be reported among women who discontinued. An alternative explanation is that women who discontinued may be more likely to provide an ex post facto rationalization, identifying side-effects after discontinuation to rationalize stopping. Longitudinal research could aid in disentangling these questions. Additionally, we did not ask non-users whether they feared any side-effects; these women may differ from those who have already started using contraception, particularly related to infertility.

Understanding the influence of experiences versus concerns, and how they relate to each other, can guide the development of clinical counseling tools and communication campaigns to help women make informed decisions about contraception. Additional research is needed to disentangle the effect of experiencing versus fearing side-effects on contraceptive use.

## Figures and Tables

**Table:** 

	**Current users**	**Recent users**
N	2020	622
	%	%
Age		
15–24	30.1	30.1
25–34	44.3	46.0
35+	25.6	23.9
Education		
None	36.6	35.5
Primary	39.9	38.7
Secondary+	23.5	25.8
Parity		
0	10.4	22.5
1	24.0	20.0
2	20.4	18.3
3	11.5	9.5
4+	33.7	29.8
Wealth		
Lowest	15.1	15.4
Middle lowest	18.4	19.4
Middle	21.4	19.8
Middle lhighest	20.0	18.0
Highest	25.1	27.4
Residence		
Urban	35.6	35.1
Rural	64.7	64.9
Method type		
Implant	33.2	18.1
IUD	2.4	1.3
Injectables	57.4	69.0
Pill	6.5	9.6
EC	0.4	2.0

## Appendix A Question to assess experienced side-effects

**What are the side-effects that you are currently experiencing?**

• Less bleeding or no bleeding
• Heavier bleeding
• Irregular bleeding/spotting
• Uterine cramping/lower abdominal pain
• Gained weight
• Lost weight
• Facial spotting
• Headaches
• Got infection
• Nausea/vomiting
• Increased menstrual cramping
• Lowered sex drive
• Decreased sexual pleasure
• Vaginal dryness
• General weakness/pain
• Diarrhea
• Partner feels during sex
• Pain at insertion site
• Mood swings
• Backache
• OTHER
• DON'T KNOW
• NO RESPONSE

## Appendix B Question to assess feared side-effects

**What are the side-effects that you ARE WORRIED ABOUT EXPERIENCING while using this method, but are not actually experiencing?**

• Less bleeding or no bleeding
• Heavier bleeding
• Irregular bleeding/spotting
• Uterine cramping/lower abdominal pain
• Weight change
• Facial spotting
• Headaches
• General weakness/pain
• Pain at insertion site
• Got infection
• Method gets lost inside body
• Nausea/vomiting
• Lowered sex drive
• Decreased sexual pleasure
• Vaginal dryness
• Partner feels during sex
• Infertility/sterility
• Delayed return to fertility
• Deformation of babies
• Diarrhea
• Cancer/fibroids
• Blood build up/impurities
• Pills accumulate in body
• Increased hair growth
• Mood swings
• OTHER
• DON'T KNOW
• NO RESPONSE
